# Fault Diagnosis Method for Rolling Bearings Based on Composite Multiscale Fluctuation Dispersion Entropy

**DOI:** 10.3390/e21030290

**Published:** 2019-03-18

**Authors:** Xiong Gan, Hong Lu, Guangyou Yang

**Affiliations:** 1 School of Mechanical and Electronic Engineering, Wuhan University of Technology, Wuhan 430070, China; 2 Institute of Agricultural Machinery, Hubei University of Technology, Wuhan 430068, China; 3 Hubei Province Engineering Technology Research Center for Intelligent Agricultural Machinery, Wuhan 430068, China

**Keywords:** CMFDE, mRMR, rolling bearings, fault diagnosis

## Abstract

This paper proposes a new method named composite multiscale fluctuation dispersion entropy (CMFDE), which measures the complexity of time series under different scale factors and synthesizes the information of multiple coarse-grained sequences. A simulation validates that CMFDE could improve the stability of entropy estimation. Meanwhile, a fault recognition method for rolling bearings based on CMFDE, the minimum redundancy maximum relevancy (mRMR) method, and the k nearest neighbor (kNN) classifier (CMFDE-mRMR-kNN) is developed. For the CMFDE-mRMR-kNN method, the CMFDE method is introduced to extract the fault characteristics of the rolling bearings. Then, the sensitive features are obtained by utilizing the mRMR method. Finally, the kNN classifier is used to recognize the different conditions of the rolling bearings. The effectiveness of the proposed CMFDE-mRMR-kNN method is verified by analyzing the standard experimental dataset. The experimental results show that the proposed fault diagnosis method can effectively classify the conditions of rolling bearings.

## 1. Introduction

The working performance of rolling bearings directly affects the safety, reliability, and stability of rotating machinery. When the rolling bearings fail, the collected vibration signals often exhibit complex nonlinear characteristics [1,2,3,4]. In recent years, time–frequency analysis methods have been exploited to decompose vibration signals by using empirical mode decomposition (EMD) [5,6], local mean decomposition (LMD) [7,8], and intrinsic timescale decomposition (ITD) [9,10]. However, these signal processing methods have some weaknesses such as end effect and mode mixing, which often fail to extract fault features effectively and reduce the fault recognition accuracy. Furthermore, mechanical fault diagnosis methods based on thermodynamic entropy [11,12,13,14] have been proposed. Nevertheless, thermodynamic data are difficult to collect online and are sensitive to the environment, which is not suitable for the early fault diagnosis of rolling bearings. Therefore, it is very necessary to study a novel fault diagnosis method for rolling bearings. With different faults, the vibration signal complexity of rolling bearings could be different. Hence, the nonlinear dynamics theory can be directly applied to extract the fault features of rolling bearings without signal decomposition or transformation.

In 1991, Pincus et al. proposed approximate entropy (APE) to measure the complexity of nonlinear time series and applied it to analyze physiological time series [15]. Subsequently, APE was utilized for extracting the fault features of rolling bearings [16]. The results show that APE can effectively characterize the types of bearings. However, APE has the disadvantage of producing inaccurate entropy values and of having a tedious computational time. Based on the research on APE, sample entropy (SE) was created by Richman et al. in 2000 [17], which could be more immune to the noise interference, and was applied in the fault diagnosis method for the rolling bearings by Han et al. [18]. However, the shortcomings of a longer calculation time and being vulnerable to mutation signal still existed in SE. In 2002, Bandt et al. [19] proposed the permutation entropy (PE), which possessed a fast computational speed, a simple calculation process, and the capacity to resist disturbance. Considering the advantages of PE, Cheng et al. [20] applied PE to the fault recognition of rolling bearings. Meanwhile, in consideration of the deficiency of single scale, multiscale sample entropy (MSE) [21] and multiscale permutation entropy (MPE) [22,23,24] were proposed, which were used to extract the fault features for rolling bearings [25] and reflect the characteristics of milling force signals [26], respectively. However, PE also failed to consider the signal amplitude. In 2016, to overcome the problems of APE, SE, and PE, Hamed Azami et al. [27] proposed a nonlinear time complexity evaluation method based on dispersion entropy (DE), which would not create unreliable entropy values as well as was insensitive to noise interference, had high computational efficiency, and could solve the equivalence problem. Thereafter, Mostafa Rostaghi et al. [28] applied DE to classify the conditions of rolling bearings and gears. In addition, Hamed Azami et al. [29] proposed fluctuation dispersion entropy (FDE), which considered the fluctuation of nonlinear time series on the basis of DE. However, FDE still had some space to be improved; for example, it only evaluated the complexity of nonlinear time series from a single scale factor. 

In this work, multiscale fluctuation dispersion entropy (MFDE) was originally proposed to analyze the complexity of nonlinear time series under various scale factors. To overcome the shortcomings of the coarsening method in the multiscale process for MFDE, the composite multiscale fluctuation dispersion entropy (CMFDE) was further proposed, which could synthesize the information of multiple coarse-grained sequences and could reduce the standard deviation (SD) of entropy estimation. Meanwhile, a novel fault diagnosis method based on CMFDE, minimum redundancy maximum relevancy (mRMR) [30], and k nearest neighbor (kNN) (CMFDE-mRMR-kNN) is developed for the rolling bearings. As the core of this method, the function of CMFDE is to extract the nonlinear fault features of rolling bearings. Then, mRMR is applied to extract the sensitive fault features to reduce feature dimension and to improve the efficiency of fault diagnosis method. Moreover, the kNN classifier is chosen to recognize the fault conditions of the rolling bearings. The experimental results show that the proposed method can effectively extract the fault features of rolling bearings and can achieve a high recognition accuracy.

## 2. FDE, MFDE, and CMFDE

### 2.1. FDE

Input: Nonlinear time series X={x(1),x(2),⋯,x(N)}, embedding dimension *m*, time delay *τ*, and number of classes *c*

Output: *FDE*(*X*,*m*,*τ*,*c*)

Step 1. X={x(1),x(2),⋯,x(N)} is mapped to Y={y(1),y(2),⋯,y(N)} by using the normal cumulative distribution function (NCDF), $y\left(j\right)=\frac{1}{\sigma \sqrt{2\pi }}\int_{-\infty }^{x\left(j\right)}e^{\frac{-\left(t-\mu \right)}{2\sigma^{2}}}dt$. Here, *μ* and *σ* are the mean and SD of X, respectively.

Step 2. *y*(*j*) is mapped to *z*(*j*), which can be defined as *z*(*j*)=*R*(*c*∙*y*(j)+0.5), where c is an integer and *R*(∙) represents the rounding function.

Step 3. For Z = {z(1),z(2), ⋯, z(N)}, with embedding dimension *m* and time delay *τ*, the embedding vector $Z^{m.\tau ,c}=\left\{Z^{m.\tau ,c}\left(1\right),Z^{m.\tau ,c}\left(2\right),\cdots ,Z^{m.\tau ,c}\left(N-\left(m-1\right)\tau \right)\right\}$ is defined as

(1) $$\{\begin{array}{c} Z^{m.\tau ,c}\left(1\right)=\left\{z\left(1\right),z\left(1+\tau \right),\cdots ,z\left(1+\left(m-1\right)\tau \right)\right\}\\ Z^{m.\tau ,c}\left(2\right)=\left\{z\left(2\right),z\left(2+\tau \right),\cdots ,z\left(2+\left(m-1\right)\tau \right)\right\}\\ \vdots \\ Z^{m.\tau ,c}\left(N-\left(m-1\right)\tau \right)=\left\{z\left(N-\left(m-1\right)\tau \right),z\left(N-\left(m-2\right)\tau \right),\cdots ,z\left(N\right)\right\} \end{array}$$

Step 4. Transform $Z^{m.\tau ,c}=\left\{Z^{m.\tau ,c}\left(1\right),Z^{m.\tau ,c}\left(2\right),\cdots ,Z^{m.\tau ,c}\left(N-\left(m-1\right)\tau \right)\right\}$ into $F^{m.\tau ,c}=\left\{F^{m.\tau ,c}\left(1\right),F^{m.\tau ,c}\left(2\right),\cdots ,F^{m.\tau ,c}\left(N-\left(m-1\right)\tau \right)\right\}$ by the following equation

(2) $$\{\begin{array}{c} F^{m.\tau ,c}\left(1\right)=\left\{z\left(1+\tau \right)-z\left(1\right)+c,\cdots ,z\left(1+\left(m-1\right)\tau \right)-z\left(1+\left(m-2\right)\tau \right)+c\right\}\\ F^{m.\tau ,c}\left(2\right)=\left\{z\left(2+\tau \right)-z\left(2\right)+c,\cdots ,z\left(2+\left(m-1\right)\tau \right)-z\left(2+\left(m-2\right)\tau +c\right)\right\}\\ \vdots \\ F^{m.\tau ,c}\left(N-\left(m-1\right)\tau \right)=\left\{z\left(N-\left(m-2\right)\tau \right)-z\left(N-\left(m-1\right)\tau \right)+c,\cdots ,z\left(N\right)-z\left(N-\tau \right)+c\right\} \end{array}$$

Step 5. $F^{m.\tau ,c}\left(j\right)=\left\{z\left(j+\tau \right)-z\left(j\right)+c,\cdots ,z\left(j+\left(m-1\right)\tau \right)-z\left(j+\left(m-2\right)\tau \right)+c\right\}$ is assigned to the dispersion pattern $\pi_{v_{0}v_{1}\cdots v_{\left(m-2\right)}}$, where $v_{0}=z\left(j+\tau \right)-z\left(j\right)+c,v_{1}=z\left(j+2\tau \right)-z\left(j+\tau \right)+c,\cdots ,v_{\left(m-2\right)}=z\left(j+\left(m-1\right)\tau \right)-z\left(j+\left(m-2\right)\tau \right)+c$. For each possible dispersion pattern $\pi_{v_{0}v_{1}\cdots v_{\left(m-2\right)}}$, its relative frequency $p\left(\pi_{v_{0}v_{1}\cdots v_{\left(m-2\right)}}\right)$ is given by

(3) $$p\left(\pi_{v_{0}v_{1}\cdots v_{\left(m-2\right)}}\right)=\frac{number\left\{j|j\leq N-\left(m-1\right)\tau ,F^{m.\tau ,c}\left(j\right){\;\mathrm{has}\;\mathrm{type}\;\pi }_{v_{0}v_{1}\cdots v_{\left(m-2\right)}}\right\}}{N-\left(m-1\right)\tau }$$

Step 6. FDE(X,m,τ,c) can be calculated by

(4) $$FDE\left(X,m,\tau ,c\right)=-\sum_{\pi =1}^{{\left(2c-1\right)}^{m-1}}p\left(\pi_{v_{0}v_{1}\cdots v_{\left(m-2\right)}}\right)\ln\left(p\left(\pi_{v_{0}v_{1}\cdots v_{\left(m-2\right)}}\right)\right)$$

### 2.2. MFDE

Input: Nonlinear time series X={x(1),x(2),⋯,x(N)}, embedding dimension *m*, time delay *τ*, number of classes *c*, and maximum scale factor *s_max_*

Output: *MFDE*

Initialization: *MFDE* = Ø and *s* = 1

Step 1. X={x(1),x(2),⋯,x(N)} is divided to $y^{s}={\left\{y^{s}\left(j\right)\right\}}_{j=1}^{\left[N/s\right]},\;\mathrm{and}\;y^{s}\left(j\right)$ is defined as

(5) $$y^{s}\left(j\right)=\frac{1}{s}\sum_{i=\left(j-1\right)s+1}^{js}x\left(i\right)$$

where j=1,2, ⋯,[N/s], [·] represents the rounding function.

Step 2. MFDE(X,m,τ,c,s) can be obtained by

(6) $$MFDE\left(X,m,\tau ,c,s\right)=FDE\left(y^{s},m,\tau ,c\right)$$

Step 3. MFDE=MFDE∪MFDE(X,m,τ,c,s), *s* = *s* + 1. Keep running Steps 1–3 if *s* is smaller than *s_max_*.

### 2.3. CMFDE

Input: Nonlinear time series X={x(1),x(2),⋯,x(N)}, embedding dimension *m*, time delay *τ*, number of classes *c*, and maximum scale factor *s_max_*

Output: *CMFDE*

Initialization: *CMFDE* = Ø and *s* = 1

Step 1. X={x(1),x(2),⋯,x(N)} is divided to $y^{s,q}={\left\{y^{s,q}\left(j\right)\right\}}_{j=1}^{[\left(N+1\right)/s]-1}$, and $y^{s,q}\left(j\right)$ is defined as

(7) $$y^{s,q}\left(j\right)=\frac{1}{s}\sum_{i=\left(j-1\right)s+q}^{js+q-1}x\left(i\right)$$

where j=1,2,⋯,[(N+1)/s]−1, q=1,2,⋯, s.

Step 2. For each scale factor *s*, calculate FDE of $y^{s,1},\;y^{s,2},$ …, $y^{s,s}$, and then, average these FDE values as CMFDE(X,m,τ,c,s), which can be obtained by

(8) $$CMFDE\left(X,m,\tau ,c,s\right)=\frac{1}{s}\sum_{q=1}^{s}FDE\left(y^{s,q},m,\tau ,c\right)$$

Step 3. CMFDE=CMFDE∪CMFDE(X,m,τ,c,s), *s* = *s* + 1. Keep running Steps 1–3 if *s* is smaller than *s_max_*.

Parameters analysis of CMFDE: If *m* is too small, CMFDE cannot accurately observe the dynamic behavior of a nonlinear time series. Conversely, if *m* is too large, CMFDE cannot detect small variations. *τ* has a small effect on CMFDE. When *c* is too large, CMFDE is sensitive to noise. When *c* is too small, two very different amplitude values are assigned to the same category. If *s_max_* is too small, CMFDE cannot fully extract the fault features of a nonlinear time series. If *s_max_* is too large, CMFDE will generate unstable entropy values. In addition, larger *m*, *c*, and *s_max_* will lead to low computational efficiency. Here, *m*, *τ*, *c*, and *s_max_* are selected as 2, 1, 5, and 20, respectively.

**Figure 1:**
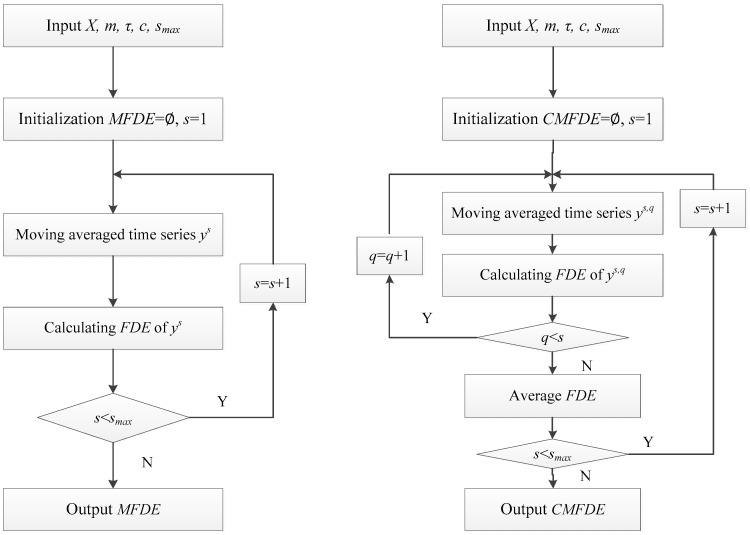
The flow charts of multiscale fluctuation dispersion entropy (MFDE) and composite multiscale fluctuation dispersion entropy (CMFDE).

Figure 1 shows the flow charts of MFDE and CFMDE.

### 2.4. Comparison between CMFDE and MFDE

**Figure 2:**
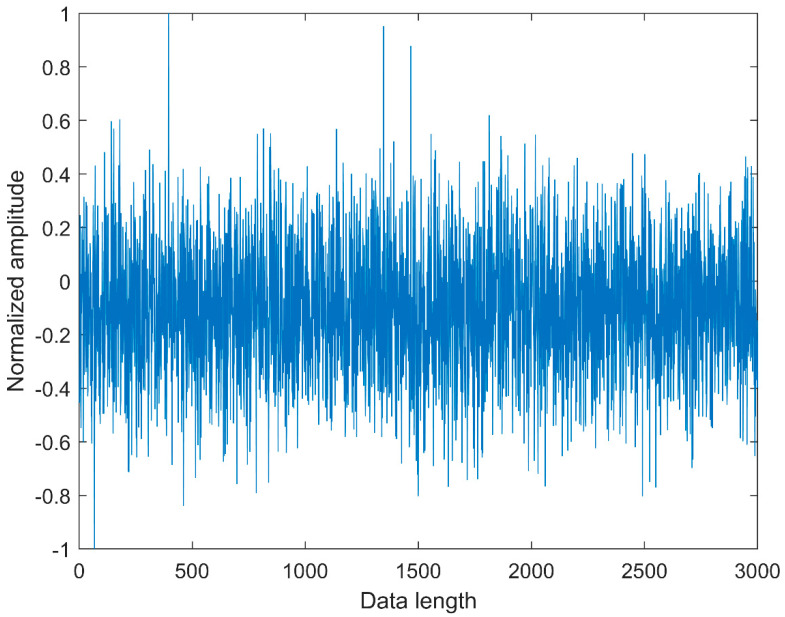
The waveform of white noise.

**Figure 3:**
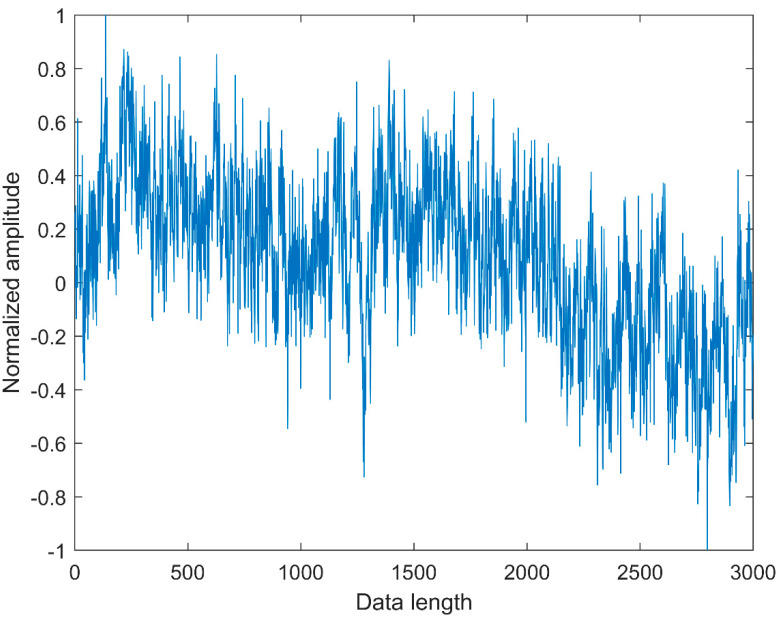
The waveform of pink noise.

**Figure 4:**
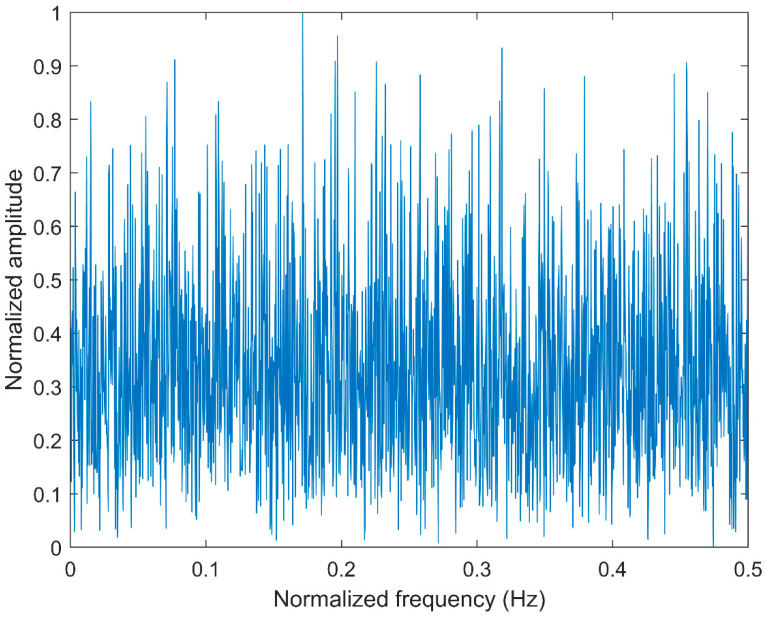
The frequency spectrum of white noise.

**Figure 5:**
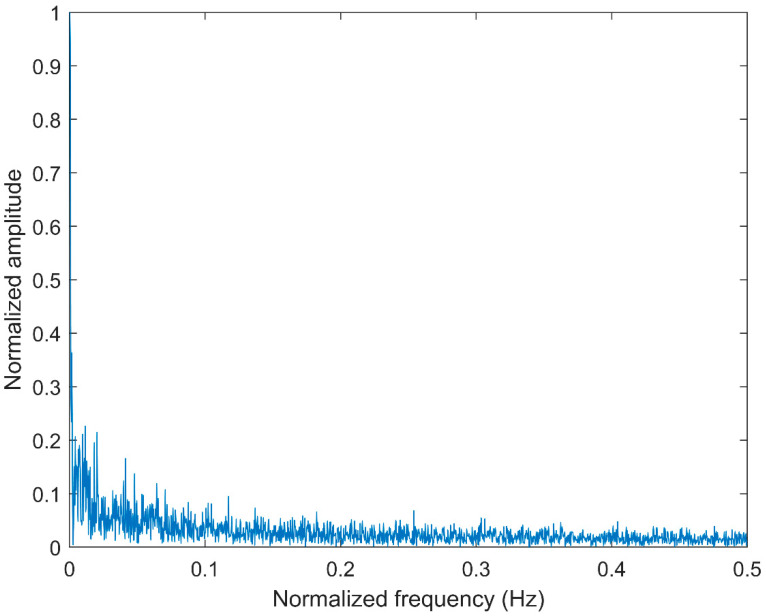
The frequency spectrum of pink noise.

**Figure 6:**
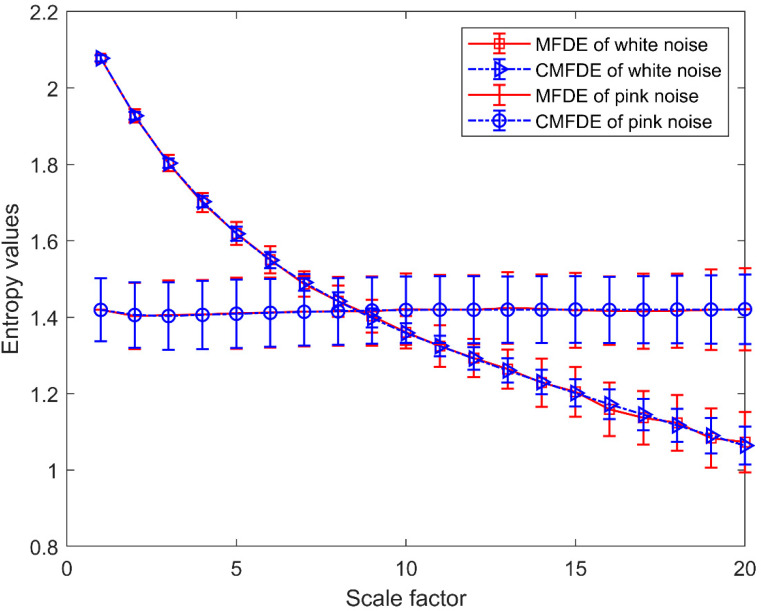
The entropy values of the two simulated signals.

To verify the advantages of CMFDE, the white noise and pink noise are adopted to perform the comparison between MFDE and CMFDE. Here, 100 groups of white noise and pink noise with data lengths of 3000 points are generated. Figure 2, Figure 3, Figure 4 and Figure 5 show the time domain waveform and frequency spectrum of two simulated signals, respectively. Figure 6 shows the mean and SD values of MFDE and CMFDE for two simulated signals. In this simulation, the embedding dimension is *m* = 2, the time delay is *τ* = 1, the number of classes is *c* = 5, and the largest number of scale factor is *s_max_* = 20. As shown in Figure 6, under the same scale factor, the difference between the mean value of MFDE and the mean value of CMFDE is unobvious. As the scale factor *s* increases, for white noise, MFDE and CFMDE decrease gradually. However, for pink noise, MFDE and CFMDE tend to be constant values. In addition, with a large-scale factor, the MFDE and CFMDE values of white noise are less than the corresponding entropy values of pink noise. The reason is that the information of white noise is mainly located in the small-scale factor and that the white noise is more irregular than the pink noise. Especially, the SD value of CMFDE is smaller than that of MFDE for both the white noise and pink noise, which indicates that CMFDE displays a better stability than MFDE for the entropy evaluation of nonlinear time series.

## 3. Fault Diagnosis Method for Rolling Bearings Based on CFMDE, mRMR, and KNN

### mRMR Feature Selection

Sequence the forward search strategy to get the sensitive features from original feature set *OF*, assume the *k* features have been chosen to set up the sensitive feature set *SF*, and choose the (*k*+1)-th feature from the remaining feature set {*OF* −*SF*} according to the following criteria

(9) $$sf_{k+1}={\mathrm{argmax}}_{of_{j}\in OF-SF}\left\{I\left(of_{j};L\right)-\frac{1}{k}\sum_{of_{i}\in OF-SF}I\left(of_{i};of_{j}\right)\right\}$$

where $I\left(of_{j};L\right)$ is the mutual information between feature *of_j_* and class *L* and where $I\left(of_{i};of_{j}\right)$ is the mutual information between feature *of_i_* and feature *of_j_*.

The concrete steps of the mRMR method can be described as follows:

Input: Original feature set $OF=\left\{of_{1},of_{2},\cdots ,of_{s}\right\}$, label information *L*, and the number of sensitive feature subset *p*

Output: Sensitive feature subset $SF=\left\{sf_{1},\;sf_{2},\cdots ,sf_{p}\right\}$

Initialization: SF = ∅ and *p* = 0

Step 1. Choose the first sensitive feature $sf_{1}$ according to $sf_{1}=argmax_{i=1,2,\cdots ,s}\left\{I\left(of_{i};L\right)\right\}$, $SF=\left\{sf_{1}\right\}$, and *k* = 1.

Step 2. Choose the (*k*+1)th sensitive feature $sf_{k+1}$ from the remaining feature set {*OF* −*SF*} according to $sf_{k+1}={\mathrm{argmax}}_{of_{j}\in OF-SF}\left\{I\left(of_{j};L\right)-\frac{1}{k}\sum_{of_{i}\in OF-SF}I\left(of_{i};of_{j}\right)\right\}$, $SF=SF\cup \left\{sf_{k+1}\right\}$, and *k* = *k* + 1.

Step 3. Keep running Step 2 until the number of sensitive feature subset |*SF*| is equal to *p*.

The CMFDE-mRMR-kNN method includes the following three steps.

Input: Input the training vibration signals of *K* different conditions $VS_{train}=\left[VS_{train}^{1},VS_{train}^{2},\cdots ,VS_{train}^{K}\right]$, testing vibration signals $VS_{test}$, number of sample points *N*, embedding dimension *m*, number of classes *c*, time delay *τ*, maximum scale factor *s_max_*, and number of sensitive features *p*

Output: Testing label *L_test_*

Step 1. Vibration signals collection and samples design: The training vibration signals *VS_train_* under each condition are divided into nonoverlapping data samples, each sample contains *N* points, each condition contains *M_train_* samples, and the training label is *L_trian_*. Meanwhile, splitting the testing vibration signals *VS_test_* into *M_test_* testing data samples without overlapping, each sample also contains *N* points.

Step 2. Feature extraction and feature selection: CMFDE method is adopted to extract the fault features and to obtain the training matrix $T_{train}$. The mRMR method is employed to choose the first *p* features to achieve the sensitive training matrix $T_{train,\mathrm{mRMR}}$. Meanwhile, according to the ranking results in the training samples, the sensitive testing matrix $T_{test,\mathrm{mRMR}}$ can be obtained.

Step 3. Pattern recognition: To classify the conditions of the rolling bearings, input $T_{train,\mathrm{mRMR}}$, $L_{trian}$, and $T_{test,\mathrm{mRMR}}$ into the kNN classifier and output the testing label $L_{test}$.

**Figure 7:**
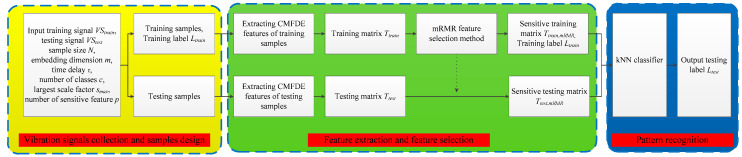
Flowchart of the CMFDE-mRMR-kNN method.

Figure 7 shows the flowchart of the CMFDE-mRMR-kNN method.

## 4. Experimental Verification and Analysis

**Figure 8:**
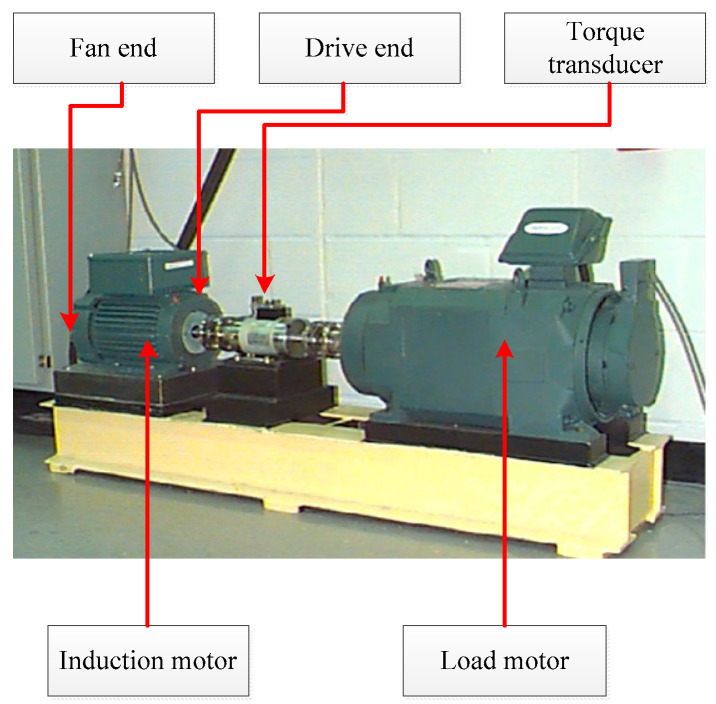
The test bench.

**Figure 9:**
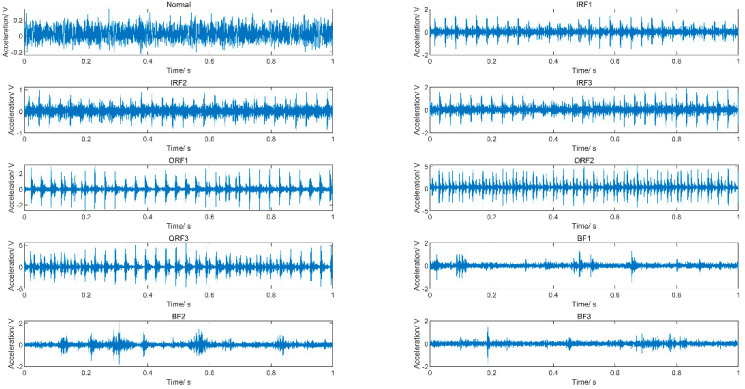
The time domain waveform under 10 different conditions.

The case university bearings dataset is utilized to validate the effectiveness of the proposed CMFDE-mRMR-kNN method. The test bench is shown in Figure 8; more system details are described in Reference [31]. The experimental bearing is 6203–2RS JEM SKF (SKF, Gothenburg, Sweden) at the fan end. The accelerometer is used for gathering the vibration signals. Vibration signals under 10 different conditions are collected, which are shown in Figure 9. The sampling frequency is 12,000 Hz. A detailed description is shown in Table 1. It is hard to recognize the specific fault condition of bearings based on the time domain waveform. Thus, the proposed CMFDE-mRMR-kNN method is employed for classifying different conditions of rolling bearings.

The vibration signals under 10 different conditions with the number of sample points *N* = 1024, the embedding dimension *m* = 2, the time delay *τ* = 1, the number of classes *c* = 5, the maximum scale factor *s_max_* = 20, and the number of sensitive features *p* = 10 are inputted. Splitting the vibration signals into multiple data samples without overlapping, each condition contains *M* = 100 data samples. For each condition, *M_train_* = 25 samples are randomly selected as the training samples, the training label was *L_trian_*, and the remaining *M_test_* = 75 samples are used as testing samples. Then, the training matrix $T_{train}\in {ℜ}^{20\times 250}$ is computed, and the first 10 features to achieve a sensitive training matrix $T_{train,mRMR}\in {ℜ}^{10\times 250}$ by adopting the mRMR method are chosen. According to the feature selection results, the sensitive testing matrix $T_{test,mRMR}\in {ℜ}^{10\times 750}$ is obtained. Finally, $T_{train,\mathrm{mRMR}}$, $L_{trian}$, and $T_{test,\mathrm{mRMR}}$ are inputted to the kNN classifier(*k* = 1), and the testing label $L_{test}$ is outputted.

**Figure 10:**
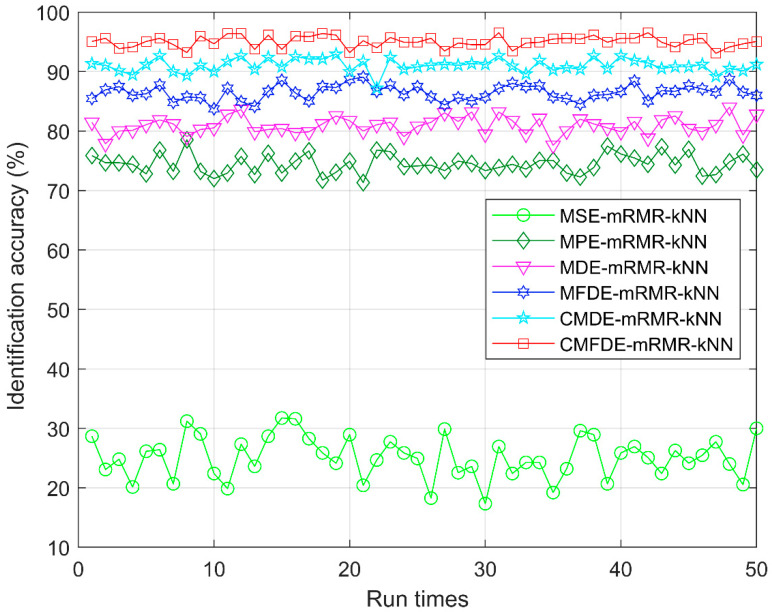
The identification accuracy with the minimum redundancy maximum relevancy (mRMR) method.

**Figure 11:**
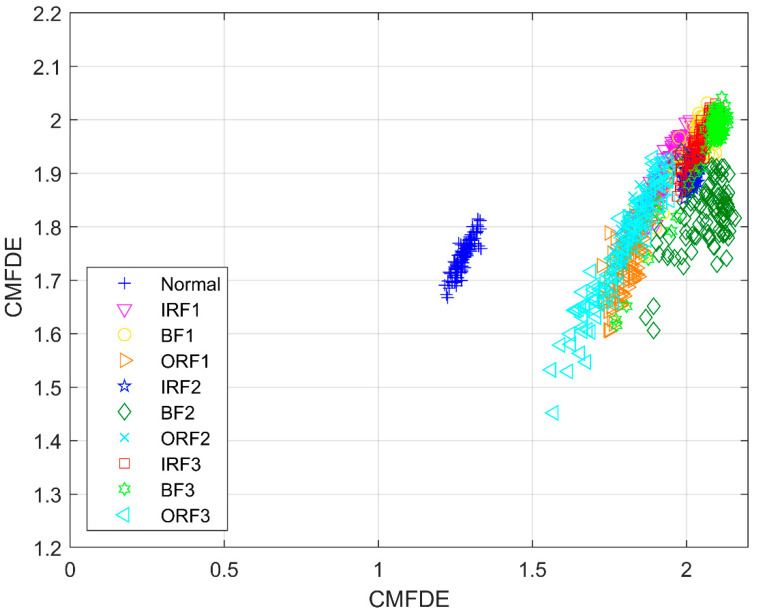
The distribution of the first two CMFDE features without using the mRMR method.

To verify the superiority of CMFDE-mRMR-kNN, the recognition accuracy of the MSE-mRMR-kNN, MPE-mRMR-kNN, MDE-mRMR-kNN, MFDE-mRMR-kNN, CMDE-mRMR-kNN, and CMFDE-mRMR-kNN are compared. For MSE-mRMR-kNN, MPE-mRMR-kNN, MDE-mRMR-kNN, MFDE-mRMR-kNN, and CMDE-mRMR-kNN, the fault features by MSE, MPE, MDE, MFDE, and CMDE are extracted, separately. Then, mRMR method is employed to choose the sensitive features. Finally, the sensitive features are input to the kNN classifier and output the testing label. For MSE-mRMR-kNN, *m* = 2, *r* = 0.15std, and *s_max_* = 20. For MPE-mRMR-kNN, *m* = 5, *τ* = 1, and *s_max_* = 20. For MDE-mRMR-kNN, *m* = 2, *τ* = 1, *c* = 5, and *s_max_* = 20. For MFDE-mRMR-kNN, *m* = 2, *τ* = 1, *c* = 5, and *s_max_* = 20. For CMDE-mRMR-kNN, *m* = 2, *τ* = 1, *c* = 5, and *s_max_* = 20. Here, *m* is the embedding dimension, *τ* is the time delay, *c* is the number of classes, *r* is the tolerance, and *s_max_* is the largest scale factor. Figure 10 and Table 2 show the identification accuracy of different fault diagnosis methods within 50 run times. In Figure 11 and Table 2, the proposed CMFDE-mRMR-kNN method attains the highest identification accuracy (96.53%–93.06%). As demonstrated, the effectiveness of the CMFDE-mRMR-kNN method is validated, and the advantage of CMFDE in extracting sensitive features is also highlighted. Then, the SD of other methods is significantly larger than that of the proposed method, which shows that the proposed CMFDE-mRMR-kNN method has a better stability.

**Figure 12:**
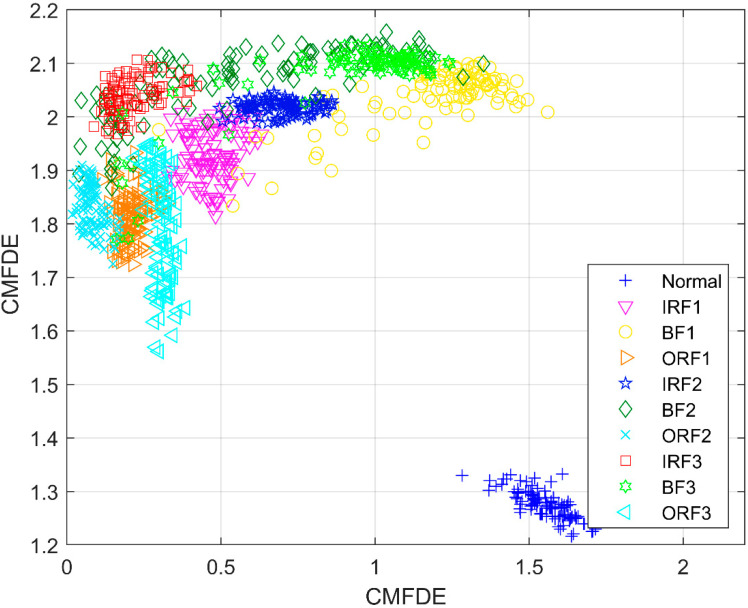
The distribution of the first two CMFDE features with the mRMR method.

**Figure 13:**
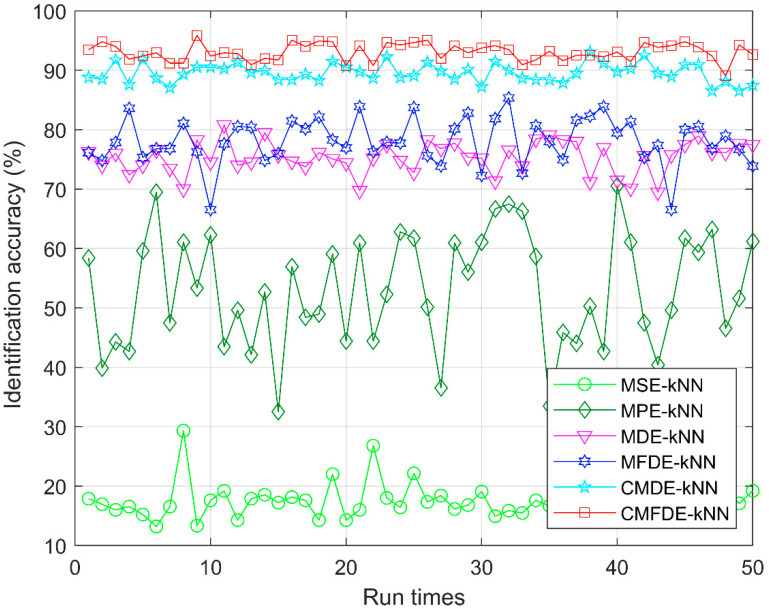
The identification accuracy without using the mRMR method.

To investigate the effectiveness of the mRMR method, Figure 11 shows the distribution of the first two initial features without using the mRMR method. Afterwards, Figure 12 shows the distribution of the first two sensitive features by employing the mRMR method. As seen in Figure 11, the clustering centers of different types are mixed together, which makes it difficult to distinguish the different conditions. However, the clustering ability in Figure 12 is superior to the ability in Figure 11, and different conditions can be easily recognized. In order to further verify the necessity of the mRMR method in MSE-mRMR-kNN, MPE-mRMR-kNN, MDE-mRMR-kNN, MFDE-mRMR-kNN, CMDE-mRMR-kNN, and CMFDE-mRMR-kNN, the first 10 scale factors of the original features are selected instead of applying the mRMR method and the new MSE-kNN, MPE-kNN, MDE-kNN, MFDE-kNN, and CMDE-kNN, CMFDE-kNN methods can be obtained. In order to reduce the impact of randomness, 50 run times are conducted under the same parameters used in Figure 10 and Table 2. The identification results are shown in Figure 13 and Table 3. As demonstrated, all the identification accuracies of the five methods are lower than that combined with the mRMR method, which verify the superiority of mRMR in selecting the sensitive features. It can be also found that the CMFDE-kNN method still achieves the highest diagnosis accuracy, which further demonstrates the advantage of the CMFDE for feature extraction.

**Figure 14:**
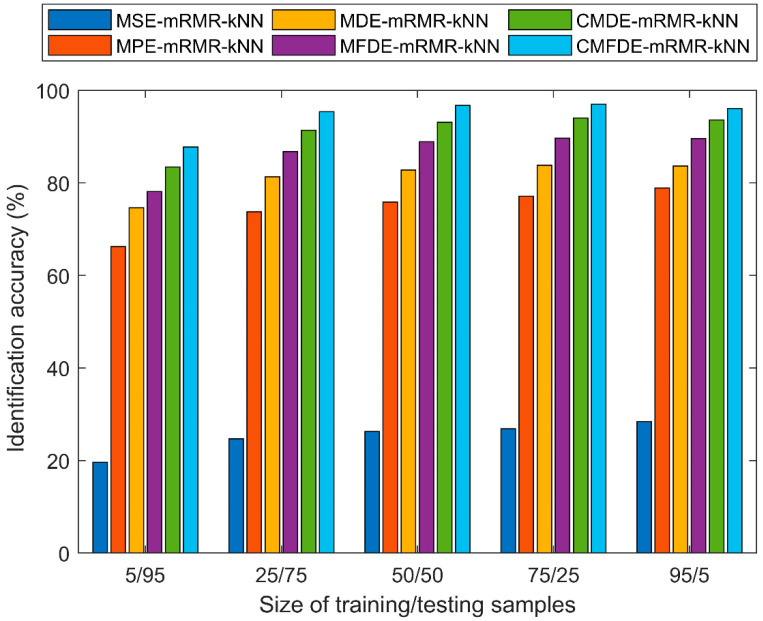
The average recognition accuracy under different sizes of training/testing samples.

To analyze the relationship between the identification accuracy and the number of training/testing samples, we divide the number of training/testing samples into five situations (5/95, 25/75, 50/50, 75/25, and 95/5) and design separately to compute the average identification accuracy of different diagnosis methods within 50 run times. Figure 14 shows the average classification accuracy under different sizes of training/testing samples. As shown in Figure 14, even though the number of training/testing samples is different, the proposed CMFDE-mRMR-kNN still obtains the highest average classification accuracy. Meanwhile, if the number of training samples is far less than that of testing samples (such as 5/95), the different methods obtain the least average identification accuracy. It can be found that, if there are sufficient training samples (such as 25/75, 50/50, 75/25, and 95/5), five different methods can achieve a higher average identification accuracy. However, excessive training samples could result in longer training computation time. Therefore, in order to achieve the balance between identification accuracy and computational efficiency, the number of training/testing samples is 25/75.

**Figure 15:**
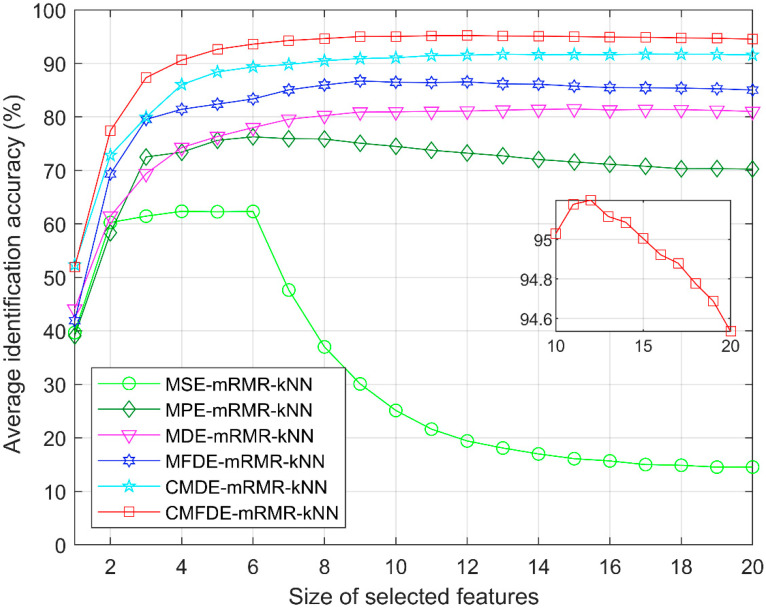
The average identification accuracy under different sizes of sensitive features.

To analyze the relationship between the identification accuracy and the size of sensitive features, an average identification accuracy can be achieved by conducting 50 run times. The corresponding average identification result is shown in Figure 15. It can be found that the CMFDE-mRMR-kNN method can achieve much a higher identification accuracy, which verifies the superiority of the proposed method. Moreover, with the increasing size of the selected features, the identification accuracy is not always increasing, a too large or too small size of sensitive fault features will lead to a decline in the identification accuracy. The reason is that a too small size of sensitive features contains less fault information. On the contrary, a too large size of sensitive features will lead to a redundancy of fault information and a reduction of the identification accuracy. For MSE-mRMR-kNN, MPE-mRMR-kNN, MDE-mRMR-kNN, MFDE-mRMR-kNN, CMDE-mRMR-kNN, and CMFDE-mRMR-kNN, the optimal sizes of sensitive features are 4, 6, 15, 9, 17, and 12, respectively. The corresponding highest average identification accuracies are 62.34%, 76.26%, 81.49%, 86.70%, 91.71% and 95.20%, respectively.

## 5. Conclusions

In this paper, a nonlinear time series complexity evaluation method based on CFMDE was proposed. Compared with MFDE, CMFDE improved the stability of the entropy evaluation. Then, a fault diagnosis method for rolling bearings based on CMFDE, mRMR, and kNN was proposed. Through analyzing a standard experimental dataset, the effectiveness of the proposed CMFDE-mRMR-kNN method was validated. Meanwhile, the superiority of CMFDE in extracting sensitive fault features was highlighted, and the necessity of mRMR feature selection was also illustrated.

## Figures and Tables

**Table 1:** The detailed descriptions for 10 different conditions.

Different Conditions	Abbreviation	Fault Diameter (mm)	Motor Load (HP)	Motor Speed (rpm)	Label
Normal	Normal	0	0	1797	1
Inner race fault	IRF1	0.1778	0	1797	2
Inner race fault	IRF2	0.3556	0	1797	3
Inner race fault	IRF3	0.5334	0	1797	4
Outer race fault	ORF1	0.1778	0	1797	5
Outer race fault	ORF2	0.3556	0	1797	6
Outer race fault	ORF3	0.5334	0	1797	7
Ball fault	BF1	0.1778	0	1797	8
Ball fault	BF2	0.3556	0	1797	9
Ball fault	BF3	0.5334	0	1797	10

**Table 2:** The identification results with the mRMR method.

Different Methods	Accuracy (%)			
	Maximum	Minimum	Mean	SD
CMFDE-mRMR-kNN	96.53	93.06	95.02	0.95
CMDE-mRMR-kNN	92.93	87.20	91.04	1.19
MFDE-mRMR-kNN	89.06	83.73	86.47	1.25
MDE-mRMR-kNN	84.00	77.60	80.93	1.43
MPE-mRMR-kNN	78.53	71.33	74.47	1.65
MSE-mRMR-kNN	31.73	17.33	25.11	3.56

**Table 3:** The identification results without using the mRMR method.

Different Methods	Accuracy (%)			
	Maximum	Minimum	Mean	SD
CMFDE-kNN	95.87	89.06	93.06	1.45
CMDE-kNN	93.06	86.53	89.55	1.56
MFDE-kNN	85.33	66.53	78.11	3.98
MDE-kNN	80.80	69.46	75.34	2.68
MPE-kNN	70.53	32.53	53.02	9.63
MSE-kNN	29.33	13.20	18.27	3.49
